# Privacy-Preserving Data Aggregation against False Data Injection Attacks in Fog Computing

**DOI:** 10.3390/s18082659

**Published:** 2018-08-13

**Authors:** Yinghui Zhang, Jiangfan Zhao, Dong Zheng, Kaixin Deng, Fangyuan Ren, Xiaokun Zheng, Jiangang Shu

**Affiliations:** 1 National Engineering Laboratory for Wireless Security, Xi’an University of Posts and Telecommunications, Xi’an 710121, China; zjf291495791@163.com (J.Z.); dkx523121943@163.com (K.D.); rfyren@163.com (F.R.); 2 Westone Cryptologic Research Center, Beijing 100070, China; 3 School of Computer Science and Technology, Xi’an University of Posts and Telecommunications, Xi’an 710121, China; xiaokzheng@163.com; 4 Department of Computer Science, City University of Hong Kong, Kowloon Tong, Hong Kong, China; jgshu2-c@my.cityu.edu.hk

**Keywords:** fog computing, Internet of Things, homomorphic encryption, privacy, data aggregation

## Abstract

As an extension of cloud computing, fog computing has received more attention in recent years. It can solve problems such as high latency, lack of support for mobility and location awareness in cloud computing. In the Internet of Things (IoT), a series of IoT devices can be connected to the fog nodes that assist a cloud service center to store and process a part of data in advance. Not only can it reduce the pressure of processing data, but also improve the real-time and service quality. However, data processing at fog nodes suffers from many challenging issues, such as false data injection attacks, data modification attacks, and IoT devices’ privacy violation. In this paper, based on the Paillier homomorphic encryption scheme, we use blinding factors to design a privacy-preserving data aggregation scheme in fog computing. No matter whether the fog node and the cloud control center are honest or not, the proposed scheme ensures that the injection data is from legal IoT devices and is not modified and leaked. The proposed scheme also has fault tolerance, which means that the collection of data from other devices will not be affected even if certain fog devices fail to work. In addition, security analysis and performance evaluation indicate the proposed scheme is secure and efficient.

## 1. Introduction

In recent years, cloud computing has developed rapidly with its advantages of ultra-large-scale storage, powerful computing power, high scalability, and low cost [1]. Any company or individual can access cloud computing servers through a payment mode [2,3,4,5,6]. At the same time, with the advancement of computer technology and the development of big data, artificial intelligence, and the Internet of Things (IoT), the demand for data interaction analysis for mass terminals has rapidly increased [7,8,9]. Under the circumstances, all data files are uploaded to the cloud for processing, which will be given cost and performance pressures to the network. Especially for IoT, it is difficult to meet the low latency requirements of real-time processing [10,11]. In 2012, Cisco proposed the concept of “fog computing” in [12] to address the high latency, the lack of support for mobility and location awareness of cloud computing. The idea is to transfer some of the storage and calculation operations on the cloud to the infrastructure device, that is, fog node, which belongs to the edge network. In other words, fog computing is an extension of cloud computing. The perfect combination of cloud and fog computing makes the network more efficient.

In the smart grid, Internet of Vehicles (IoV), smart home [13], smart health [14] and other IoT application scenarios [15,16,17,18], these hybrid IoT devices’ data can be sent to the control center through fog nodes. The data often contains user’s privacy [19,20,21,22,23,24]. For instance, heart monitors are related to the life safety of each user [25], and smart meters in smart grid collect power data which reflects users’ daily lives [26,27,28,29]. In smart grid, a power company collects smart meter’s data and analyzes them to ensure that the power system runs efficiently [30].

Because the fog nodes are deployed at the edge of the network and low-traffic nodes, they are more vulnerable to hackers. Once user’s information is leaked, it will have a bad influence [31], and the sensitive data must be encrypted before uploading [32,33]. In addition, when a large number of IoT device data is transmitted to fog nodes, it will not only affect the requirements for a real-time response of IoT, but also cause problems such as network congestion. At this time, the data aggregation based on homomorphic encryption applied to fog devices is particularly important [34,35,36,37]. Specifically, when the fog device sends the aggregated data instead of the data of each IoT device to the control center, the communication overhead will be greatly reduced, although the security and privacy issues are needed to be addressed [38]. In fact, data aggregation technology is widely used in various communication networks to save bandwidth [39,40,41,42,43,44].

In this paper, we propose a privacy-preserving data aggregation scheme based on homomorphic encryption in fog computing (PDAF). At a time slot, each IoT device will report its sensing data to a fog node after the data is blinded by two secret keys and a blinding factor. The data is then collected by the control center so that the entire IoT network runs efficiently. These blinded data are aggregated by fog devices at the edge of the network. All the aggregated values are sent by the fog device to the control center, and the fog device is able to detect faulty IoT devices. Upon receiving the packet, the control center can generate relevant secret keys and get the total amount of IoT devices’ sensing data at each time slot from the aggregated blinded data. For the sake of security, packets transmitted during the communication process should be verified. In the PDAF system, only the control center can know the total amount of IoT devices’ sensing data at each time slot, and individual IoT device data is hidden. Our security and privacy analysis indicates that PDAF is secure against false data injection attacks and data modification and it can protect data privacy. Extensive evaluations show that PDAF is very efficient in terms of the computation and communication cost.

### 1.1. Related Work

In 2004, the International Telecommunication Union (ITU) expanded the concept of the IoT: Interconnections at any time, anywhere, arbitrary objects, ubiquitous networks and ubiquitous computing [45]. Cisco [12] pointed that the Internet of Things as a delay-sensitive application, which requires high real-time performance. In the era of the Internet of Everything, a new platform called fog computing is needed to support it. The author considered fog computing as a new application and service, and that there is a fruitful interaction between cloud and fog, especially in data management and analysis. In simple terms, the fog is a cloud close to the ground.

In IoT, in order to reduce communication costs, it is essential to aggregate individual IoT device’s data at associated fog device. In the previous researches, some privacy-preserving data aggregation schemes [10,26,27,30,37,46] are related to our PDAF scheme. In addition, blockchain technologies have been used for realizing fair payment in cloud computing and fog computing [47,48,49]. Zhang et al. [27] designed a privacy-preserving communication and power injection scheme over vehicle networks and 5G smart grid slice based on the Paillier encryption. In the scheme, a novel aggregation technique called hash-then-homomorphic is used to aggregate the blinded bids of different time slots. Mahmoud et al. [30] adopted two data different aggregation schemes using point addition and homomorphic encryption. Shen et al. [37] proposed a privacy-preserving multilevel user’s data aggregation and control scheme, it extended the previous one-dimensional data aggregation to two dimensions and is more suitable for practical application environments. Zhou et al. [46] also proposed a multidimensinal data aggregation scheme and is fault-tolerant. However, Zhang et al. [26] considered the EPPI scheme based on point addition is safer and more efficient. In fact, EPPI guarantees that privacy will not be leaked even if all entities in the actual application scene are dishonest. But the EPPI scheme is not fault-tolerant. Although the above schemes are suitable for fog computing-enhanced IoT, they cannot aggregate all hybrid IoT devices’ data into a single ciphertext. For fog computing-enhanced IoT, Lu et al. [10] designed a lightweight privacy-preserving data aggregation scheme which is secure and fault-tolerant, but the third-party trusted authority in this system will inevitably increase the communication overhead of the system. Different from the above schemes, in PDAF, the third-party trusted authority is not needed and data privacy is still preserved. We use the modified Paillier encryption to enable the fog device to aggregate hybrid IoT devices’ data into a single ciphertext and keep it fault-tolerant.

### 1.2. Our Contribution

In PDAF, we have made improvements based on the Paillier homomorphic encryption scheme, each IoT device can generate two secret key and a blinding factor to mask its sensitive data, and it sends the masked data to the related fog node based on wireless network. Upon receiving packets from all hybrid IoT devices, the control center can only obtain the total within the limited range instead of directly reading data of a single IoT device. Because of the blinding factor, the control center also can correctly decrypt the aggregated data in the event that an IoT device fails to send messages to the fog device. In fact, the proposed PDAF scheme is fault-tolerant. In the second place, the PDAF scheme realizes privacy protection. We notice that the fog device and the control center are curious about the sensitive data to be reported by a single IoT device or the aggregated data by the fog device. In PDAF, the attacker will not get any privacy about the user, nor can it forge or change the ciphertext to be sent to the fog device. In addition, an efficient batch verification method is adopted in order to verify the signatures of multiple users instead of verifying one by one and the computation overhead of the fog device is reduced.

### 1.3. Organization

The remaining of this paper is organized as follows. In Section 2, we introduce our system model and review some preliminary knowledge. Then, we describe the proposed PDAF scheme in detail in Section 3. Next, we give the security and privacy analysis of the proposed PDAF scheme in Section 4, followed by performance evaluation in Section 5. Finally, in Section 6, we draw our conclusions.

## 2. Models and Security Requirements

In this section, we formalize our system model, adversary model, security requirements and design goal, and give a brief review on preliminary knowledge which will serve as the building blocks of the proposed PDAF scheme.

### 2.1. System Model

**Figure 1:**
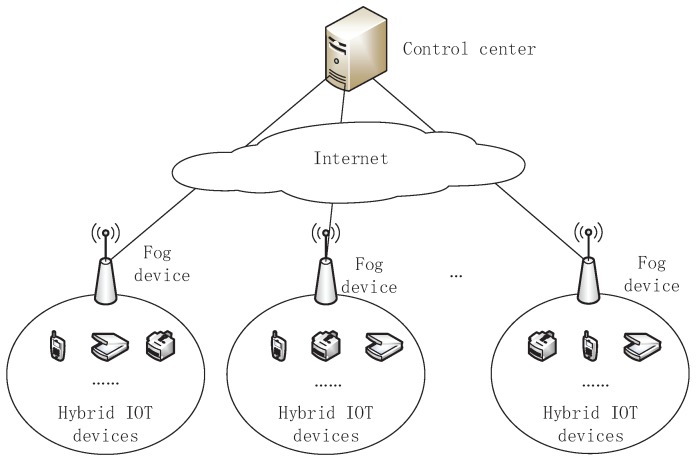
The system model of PDAF.

As shown in Figure 1, the considered system model of PDAF includes a control center, some fog devices at the network edge, and some hybrid IoT devices, which each hybrid IOT device involves a set of heterogeneous IoT devices $U=\{HID_{1},HID_{2},\ldots ,HID_{n}\}$.

- Control center. During communication, the control center generates system parameters and is responsible for registration of fog devices and IoT devices. It also collects all IoT devices data $\left(m_{1},m_{2},\ldots ,m_{n}\right)$ via fog devices periodically and analyzes the data replied by fog devices. Please note that CC cannot directly get $m_{i}\left(1\leq i\leq n\right)$ which containing the user’s privacy. In addition, when an IoT device fails to send a message, it is also necessary to make the aggregation of other users’ information unaffected. where, the control center communicates with the IoT devices via the Internet network.
- Fog devices. A fog device is also a fog node and is the most critical part of the fog computing between the hybrid IoT devices and the control center. Fog devices can be memory routers, small servers or smart phones that are deployed at the edge network. In PDAF, the fog device will forward data packets from the control center to IoT devices in their jurisdictions, aggregate all IoT devices’ data, and discover faulty IoT devices and report to control center for countermeasures.
- Hybrid IoT devices. With sensing and communication capabilities, the IoT devices HID${}_{i}$ (i=1,2,…,n) are deployed at an area in need and enable to periodically report its sensing result $m_{i}$ to control center through the relevant fog device.

### 2.2. Adversary Model

In the proposed PDAF scheme, we assume all the entities are “honest-but-curious”. More specifically, they can legitimately do their assigned tasks, but are also curious about the privacy of IoT devices, such as the control center that can intercept data from a single IoT device to gain private information about the device owner and other financial benefits information. Please note that although the entities are “curious”, they cannot collude. Similarly, each IoT device also wants to know the data of other IoT devices to determine if it is profitable. In addition, certain IoT devices may fail and stop to report for some time. Here, we assume that each IoT device can only send packets within this fog computing coverage area. It is also possible that an attacker resides between an IoT device and the control center and tries to establish two scert keys such that the IoT device and the control center seems to communication directly. In addition, some IoT attackers and outsiders are also interested in other sensitive information in the fog computing. In PDAF, we focus on the privacy-preserving data aggregation, in which false data injection attacks and data modification can be prevented.

### 2.3. Security Requirements and Design Goal

Considering the IoT and fog computing practical application environment, in order to prevent from these attackers getting sensitive data of IoT devices, our scheme should meet the following security requirements:

(1) Privacy Protection. Even if the attacker intercepts the communication data transmitted on the insecure channel, it cannot obtain the sensitive data of the IoT devices. The control center can decrypt the aggregated data but cannot get the individual information of a single device.
(2) Non-Repudiation and Unforgeability. The control center and the fog devices can verify the received data packets to ensure that the data packets come from the legal unit and has not been tampered, that is, the proposed scheme can defeat the false injection attack and detect the malicious attack. Besides, the adversary should not impersonate the control center, the fog devices, or the IoT devices.

Under the considered system model and security requirements, our design goal is to propose a privacy-preserving data aggregation scheme based on homomorphic encryption in fog computing. First, private data of IoT devices cannot be compromised. Second, the proposed scheme should be fault-tolerant. When certain IoT devices fail to work, they should be detected by the associated fog device and reported to the control center. Third, the control center and the fog device are able to authenticate the received packets to make sure that the packets have not been modified during the transmission and are really from legal IoT devices. Finally, if the proposed scheme effectively reduces the amount of channel transmission and improves the data processing efficiency of each entity, then the proposed scheme will be more practical.

## 3. Proposed PDAF Scheme

**Figure 2:**
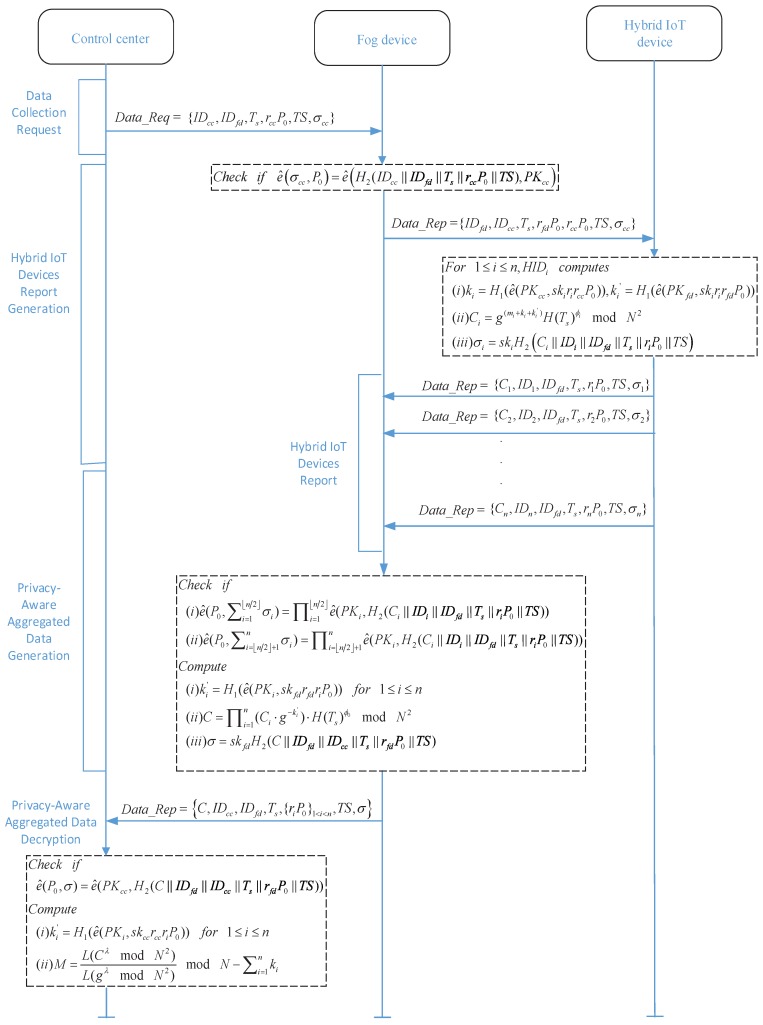
Six phases of PDAF.

In this section, we propose a privacy-preserving data aggregation scheme based on homomorphic encryption in fog computing, which consists of the following parts: preliminaries, system initialization, data collection request, hybrid IoT devices report, privacy-preserving aggregated data generation, privacy-preserving aggregated data decryption, and fault tolerance mechanism. Figure 2 summarizes the six phases of the proposed scheme. The details are given in the following:

### 3.1. Preliminaries

In this subsection, we give a brief review of bilinear pairings and the Paillier encryption algorithm.

#### 3.1.1. Bilinear Pairings

Let $G_{1}$, $G_{2}$ be a cyclic addition group and a cyclic multiplication group of prime order *q* and $P_{0}\in G_{1}$ be a generator. We call *ê* a bilinear pairing if *ê*: $G_{1}\times G_{1}\to G_{2}$ is a map with the following properties:

(1) Bilinear: For all $a,b\in Z_{q}^{*}$, *ê*$(aP_{0},bP_{0})=$ *ê*${\left(P_{0},P_{0}\right)}^{ab}$.
(2) Non-degenerate: *ê*$\left(P_{0},P_{0}\right)\neq 1_{G_{1}}$.
(3) Computable: For all $P_{0},Q\in G_{1}$, there is an efficient algorithm to compute *ê*$\left(P_{0},Q\right)$.

#### 3.1.2. Paillier Encryption Algorithm

Paillier encryption is a homomorphic encryption algorithm that consists of three algorithms: key generation, encryption, and decryption. The special as follow:

- Key Generation: Given a safety parameter κ, choose two large primes *p* and *q*, where ∣p∣=∣q∣=κ, compute N=pq and λ=lcm(p−1,q−1), define the function $L\left(u\right)=\frac{u-1}{N}$, select the generator $g\in Z_{N^{2}}^{*}$ and get the public key pk=(N,g) and the secret key λ.
- Encryption: Given a message $M\in Z_{N}$, a random number $r\in Z_{N}^{*}$ and calculate the ciphertext $C=g^{M}\cdot r^{N}\;mod\;N^{2}$.
- Decryption: Given ciphertext $C\in Z_{N^{2}}^{*}$, the corresponding plaintext is $M=\frac{L(C^{\lambda }modN^{2})}{L(g^{\lambda }modN^{2})}\;mod\;N$.

### 3.2. Details of PDAF

#### 3.2.1. System Initialization

(1) System parameters generated: In the system parameters generation stage, the control center (CC) selects the security parameter κ and generates $(q,P_{0},G_{1},G_{2},$*ê*) by running gen(κ). Then, CC selects *g* as a generator of $Z_{N^{2}}^{*}$, the security parameter $\kappa_{1}$ and two safe large prime numbers p,q. Computing a homomorphic encryption public key pair $\left(N=p_{1}q_{1},g\right)$ and the corresponding private key $\lambda =lcm(p_{1}-1,q_{1}-1)$. Next, CC defines a function $L\left(x\right)=\frac{x-1}{N}$ and chooses five secure cryptographic hash functions, $H:{\left\{0,1\right\}}^{*}\to \;Z_{N}^{*}$, $H_{1}:G_{2}\to Z_{q}^{*}$, $H_{2}:{\left\{0,1\right\}}^{*}\to G_{1}$, $H_{3}:{\left\{0,1\right\}}^{*}\to Z_{q}^{*}$, $H_{4}:G_{1}\to \;Z_{q}^{*}$ and a random element $sk_{cc}$ as its secret key and calculates $PK_{cc}=sk_{cc}P_{0}$ as its public key. Finally, CC publishes the public parameters {$(q,P_{0},G_{1},G_{2},$*ê*$,N,H,H_{1},H_{2},H_{3},H_{4}$}.
(2) Registration:
  - Fog Devices Registration.
    The fog device (FD) chooses a random element $sk_{fd}$ as its secret key and calculates $PK_{fd}=sk_{fd}P_{0}$ as its public key. Choosing random number $x\in Z_{q}^{*}$ and calculating $\alpha =H_{3}\left(x∥ID_{fd}\right)P_{0}$, $\beta =H_{3}\left(x∥ID_{fd}\right)-sk_{fd}H_{4}\left(\alpha \right)\;mod\;q$, where, $ID_{fd}$ is the identity of the fog device. Then, FD sends the parameters {$PK_{fd},\alpha ,\beta ,ID_{fd}$} to CC. After receiving the parameters {$PK_{fd},\alpha ,\beta ,ID_{fd}$}, CC verifies whether the equation $\alpha =\beta P_{0}+H_{4}\left(\alpha \right)PK_{fd}$ holds. If passed, CC publishes the public parameters {$PK_{fd},ID_{fd}$}, otherwise, refused to register.
  - Hybrid IoT Devices Registration. HID${}_{i}\left(i=1,2,\ldots ,n\right)$ chooses a random element $sk_{i}$ as its secret key and calculates $PK_{i}=sk_{i}P_{0}$ as its public key. HID${}_{i}$ Chooses random number $x_{i}\in Z_{q}^{*}$ and calculates $\alpha_{i}=H_{3}\left(x_{i}∥ID_{i}\right)P_{0}$, $\beta_{i}=H_{3}\left(x_{i}∥ID_{i}\right)-sk_{i}H_{4}\left(\alpha_{i}\right)\;mod\;q$, where, $ID_{i}$ is the identity of the hybrid IoT device. Then, HID${}_{i}$ sends the parameters {$PK_{i},\alpha_{i},\beta ,ID_{i}$} to CC. After receiving the parameters {$PK_{i},\alpha_{i},\beta ,ID_{i}$}, CC verifies whether the equation $\alpha_{i}=\beta_{i}P_{0}+H_{4}\left(\alpha_{i}\right)PK_{i}$ holds. If passed, CC publishes the public parameters {$PK_{i},ID_{i}$}, otherwise, refused to register.
(3) Blinding Factor Generated: After completing the registration, CC runs pseudo-random generator and generates *n* random numbers $\phi_{i}\in Z_{N}$ as a blinding factor for HID${}_{i}$ under each FD region and computers $\phi_{0}=-\left(\phi_{1}+\phi_{2}+\ldots +\phi_{n}\;mod\;N\right)$ as FD’s blinding factor. Please note that $\phi_{i}$ and $\phi_{0}$ are satisfied $\sum_{i=0}^{n}\phi_{i}\equiv 0\;mod\;N$. Then, CC sends $\phi_{0}$ to the registered FD, and sends $\phi_{i}$ to the registered HID${}_{i}$.

#### 3.2.2. Data Collection Request

In PDAF, the control center can collect data from related fog devices during every time slot $T_{s}$. To be specific, CC sends data collection request (*Data_Req*) packet that contains parameters {$ID_{cc},ID_{fd},T_{s},r_{cc}P_{0},TS,\sigma_{cc}$} to fog devices. Where, $ID_{cc}$ and $ID_{fd}$ is the identity of the control center and fog device respectively. Please note that $r_{cc}\in Z_{q}^{*}$ is a random number, each IOT device uses the secret key $r_{cc}P_{0}$ to establish a one-time key shared with the control center. Timestamp *TS* and $\sigma_{cc}=sk_{cc}H_{2}(ID_{cc}∥ID_{fd}∥T_{s}∥r_{cc}P_{0}∥TS)$ will be used for verifying by the fog devices. Then, the fog device runs the following steps after receiving the *Data_Req* packet:

(1) According to the difference between the current time and the timestamp TS, FD checks the freshness of *Data_Req* packet.
(2) FD verifies the signature by computing if *ê* $(\sigma_{cc},P_{0})=$*ê*$(H_{2}(ID_{cc}∥ID_{fd}∥T_{s}∥r_{cc}P_{0}∥TS),PK_{cc})$ holds.
(3) If the above equation holds, FD randomly chooses $r_{fd}\in Z_{q}^{*}$, calculates $r_{fd}P_{0}$, puts $r_{fd}P_{0}$ in the packet *Data_Req*, and broadcasts the packet that contains parameters {$ID_{fd},ID_{CC},T_{s},r_{fd}P_{0},r_{cc}P_{0},TS,\sigma_{cc}$} in its area. Please note that $r_{fd}P_{0}$ is used by hybrid IOT device HID${}_{i}$ covered by the fog device in establishing a one-time key shared with the fog device.

#### 3.2.3. Hybrid IoT Devices Report Generation

After receiving the packet *Data_Req*, hybrid IoT device HID${}_{i}$ will report its sensing data $m_{i}$ to fog device at time slot $T_{s}$. Specific steps are as follows:

(1) The hybrid IoT device HID${}_{i}$ chooses $r_{i}\in z_{q}^{*}$, computers $r_{i}P_{0}$ which is used by ID${}_{fd}$ in establishing a shared one-time key between itself and the related fog device.
(2) HID${}_{i}$ computes two shared keys as $k_{i}=H_{1}($*ê*$\left(PK_{cc},sk_{i}r_{i}r_{cc}P_{0})\right)$, $k_{i}^{\prime }=H_{1}($*ê*$\left(PK_{fd},sk_{i}r_{i}r_{fd}P_{0})\right)$, which will be used for hiding HID${}_{i}$’s sensing data $m_{i}$.
(3) HID${}_{i}$ masks its sensing data $m_{i}$ and computes ciphertext $C_{i}$ and signature $\sigma_{i}$, where
  $$C_{i}=g^{m_{i}+k_{i}+k_{i}^{\prime }}H{\left(T_{s}\right)}^{\phi_{i}}\;mod\;N^{2},$$
  $$\sigma_{i}=sk_{i}H_{2}(C_{i}∥ID_{i}∥ID_{fd}∥T_{s}∥r_{i}P_{0}∥TS).$$
  Then HID${}_{i}$ sends data collection reply *Data_Rep* packet that contains parameters {$C_{i},ID_{i},ID_{fd},T_{s},r_{i}P_{0},TS,\sigma_{i}$} to fog devices.

#### 3.2.4. Privacy-Preserving Aggregated Data Generation

Upon receiving the *Data_Rep* packet, the fog device runs the following steps:

(1) FD verifies *n* *Data_Rep* packets received to ensure that the packets are valid and have not been tampered or forged during communication. To improve the verification efficiency, FD randomly divides the *Data_Rep* packet set $S=(ID_{i},ID_{fd},T_{s},r_{i}P_{0},TS,\sigma_{i})$ (i=1,2,…,n). From *S*, ⌊n/2⌋ *Data_Rep* packets are randomly selected to form the first subset $S_{1}$, and the remaining ⌈n/2⌉ *Data_Rep* packets constitute the second subset $S_{2}$. For ease of description, suppose $S_{1}$ contains the first ⌊n/2⌋ *Data_Rep* packets and $S_{2}$ contains the second ⌈n/2⌉ *Data_Rep* packets. For IOT devices, the *Data_Rep* packets in $S_{1}$ are valid if the following equation holds, otherwise the packets are invalid.
  $$ê\left(P_{0},\sum_{i=1}^{\left\lfloor n/2\right\rfloor }\sigma_{i}\right)=\prod_{i=1}^{\left\lfloor n/2\right\rfloor }ê(PK_{i},H_{2}(C_{i}∥ID_{i}∥ID_{fd}∥T_{s}∥r_{i}P_{0}∥TS).$$
  Note that using the above verification method, the number of bilinear pairs can be reduced from 2⌊n/2⌋ to ⌊n/2⌋+1. Similarly, FD verifies the following equation. If it holds, the number of bilinear pairs also drops from 2⌊n/2⌋ to ⌊n/2⌋+1.
  $$\begin{array}{c} ê\left(P_{0},\sum_{i=\lfloor n/2\rfloor +1}^{n}\sigma_{i}\right)=\prod_{i=\lfloor n/2\rfloor +1}^{n}ê(PK_{i},H_{2}(C_{i}∥ID_{i}∥ID_{fd}∥T_{s}∥r_{i}P_{0}∥TS). \end{array}$$
(2) If the step 1 is verified, the fog device calculates
  $$\begin{array}{c} k_{i}^{\prime }=H_{1}\left(ê\left(PK_{i},sk_{fd}r_{fd}r_{i}P_{0}\right)\right)=H_{1}\left(ê\left(PK_{fd},sk_{i}r_{i}r_{fd}P_{0}\right)\right). \end{array}$$
  Then, It runs the following data aggregation operations and get the aggregate ciphertext *C* and the corresponding signature σ, the specific process are as follows:
  $$\begin{array}{ccc} C & = & \prod_{i=1}^{n}\left(C_{i}\cdot g^{-k_{i}^{\prime }}\right)\cdot H{\left(T_{s}\right)}^{\phi_{0}}\;mod\;N^{2}\\ & = & \prod_{i=1}^{n}\left(g^{m_{i}+k_{i}+k_{i}^{\prime }}\cdot g^{-k_{i}^{\prime }}\right)\cdot H{\left(T_{s}\right)}^{\phi_{0}}\;mod\;N^{2}\\ & = & g^{\sum_{i=1}^{n}\left(m_{i}+k_{i}\right)}\cdot H{\left(T_{s}\right)}^{\sum_{i=0}^{n}\phi_{i}}\;mod\;N^{2}\\ & = & g^{\sum_{i=1}^{n}\left(m_{i}+k_{i}\right)}\cdot H{\left(T_{s}\right)}^{\beta N}\;mod\;N^{2},\\ \sigma & = & sk_{fd}H_{2}(C∥ID_{fd}∥ID_{cc}∥T_{s}∥r_{fd}P_{0}∥TS). \end{array}$$
  where, because $\sum_{i=0}^{n}\phi_{i}=0\;mod\;N$, $\sum_{i=0}^{n}\phi_{i}=\beta N$.
(3) The fog device sends the *Data_Rep* packet that contains parameters {$C,ID_{cc},ID_{fd},T_{s},{\left\{r_{i}P_{0}\right\}}_{1<i<n},TS,\sigma$} to control center.

#### 3.2.5. Privacy-Preserving Aggregated Data Decryption

Upon receiving the fog device reply packet *Data_Rep*, CC first verifies the *Data_Rep* to ensure the packets’ authenticity and integrity according to the following equation:

$$ê\left(P_{0},\sigma \right)=ê(PK_{cc},H_{2}(C∥ID_{fd}∥ID_{cc}∥T_{s}∥r_{fd}P_{0}∥TS).$$

If it does hold, then CC calculates

$$\begin{array}{c} k_{i}=H_{1}\left(ê\left(PK_{i},sk_{cc}r_{cc}r_{i}P_{0}\right)\right)=H_{1}\left(ê\left(PK_{fd},sk_{i}r_{i}r_{cc}P_{0}\right)\right). \end{array}$$

Finally, it uses the private key λ to decrypt the aggregated ciphertext *C* by calculating

$$\begin{array}{ccc} M & = & \frac{L(C^{\lambda }\;mod\;N^{2})}{L(g^{\lambda }\;mod\;N^{2})}\;mod\;N-\sum_{i=1}^{n}k_{i}\\ & = & \sum_{i=1}^{n}m_{i}. \end{array}$$

#### 3.2.6. Fault Tolerance Mechanism

If some hybrid IoT devices breakdown, FD will not receive *n* *Data_Rep* packets. Then this phenomenon will directly affect the main features of the blinding factor, and $\sum_{i\in U_{i}/U_{i}^{\prime }}\phi_{i}+\phi_{0}\neq 0\;mod\;N$, which will affect the correctness of the final data decryption. Where, U${}_{i}$ means the set of all legitimate hybrid IoT devices and $U_{i}^{\prime }$ means the set of failed hybrid IoT devices ($U_{i}^{\prime }$ ∈ U${}_{i}$).

FD needs to send the set $U_{i}^{\prime }$ to control center. After receiving the set $U_{i}^{\prime }$, CC computes $H^{\prime }\left(T_{s}\right)=H{\left(T_{s}\right)}^{\sum_{i\in U_{i}^{\prime }}\phi_{i}}$ and replies to FD. After receiving $H^{\prime }\left(T_{s}\right)$, computing

$$\begin{array}{ccc} C^{\prime } & = & H^{\prime }\left(T_{s}\right)\cdot \prod_{i\in U_{i}/U_{i}^{\prime }}\left(C_{i}\cdot g^{-k_{i}^{\prime }}\cdot H{\left(T_{s}\right)}^{\phi_{0}}\right)\;mod\;N^{2}\\ & = & H{\left(T_{s}\right)}^{\sum_{i\in U_{i}^{\prime }}\phi_{i}}\cdot \prod_{i\in U_{i}/U_{i}^{\prime }}\left(g^{m_{i}+k_{i}+k_{i}^{\prime }}\cdot g^{-k_{i}^{\prime }}\cdot H{\left(T_{s}\right)}^{\phi_{0}}\right)\;mod\;N^{2}\\ & = & \prod_{i\in U_{i}/U_{i}^{\prime }}g^{\left(m_{i}+k_{i}\right)}\cdot H{\left(T_{s}\right)}^{\sum_{i\in U_{i}^{\prime }}\phi_{i}+\sum_{i\in U_{i}/U_{i}^{\prime }}\phi_{i}+\phi_{0}}\;mod\;N^{2}\\ & = & g^{\sum_{i\in U_{i}/U_{i}^{\prime }}\left(m_{i}+k_{i}\right)}\cdot H{\left(T_{s}\right)}^{\sum_{i=0}^{n}\phi_{i}}\;mod\;N^{2}\\ & = & g^{\sum_{i\in U_{i}/U_{i}^{\prime }}\left(m_{i}+k_{i}\right)}\cdot H{\left(T_{s}\right)}^{\beta N}\;mod\;N^{2}. \end{array}$$

At this time, in aggregated data decryption stage, CC uses the private key λ to decrypt the aggregated ciphertext *C* by calculating.

$$\begin{array}{ccc} M^{\prime } & = & \frac{L(C^{\prime \lambda }\;mod\;N^{2})}{L(g^{\lambda }\;mod\;N^{2})}\;mod\;N-\sum_{i\in U_{i}/U_{i}^{\prime }}k_{i}\\ & = & \sum_{i\in U_{i}/U_{i}^{\prime }}m_{i}. \end{array}$$

## 4. Security and Privacy Analysis

In this section, we give the security and privacy analysis of the proposed PDAF scheme.

### 4.1. Privacy Protection

Based on the Paillier encryption algorithm, in the hybrid IoT devices report generation stage, the sensitive data $m_{i}$ was blinded and the secret key $k_{i}$ and $k_{i}^{\prime }$ were added in the Paillier encryption algorithm to get the ciphertext $C_{i}=g^{m_{i}+k_{i}+k_{i}^{\prime }}H{\left(T_{s}\right)}^{\phi_{i}}\;mod\;N^{2}$, HID${}_{i}$ sends $C_{i}$ to the associated gateway instead of $m_{i}$ directly. Without the private key, it is infeasible to decrypt ciphertexts. Even if the adversary gets the data packet sent by tapping the wireless IoT device or the wireless communication channel, without knowing $k_{i}$, $k_{i}^{\prime }$ and λ, the adversary cannot know the sensitive data $m_{i}$ because of these secret keys cannot be computed. Despite the control center has the secret key $k_{i}$ and λ, it cannot get $k_{i}^{\prime }$, and thus cannot decrypt $C_{i}$ to recover $m_{i}$. Similarly, the fog device is also unable to read sensitive data $m_{i}$ without $k_{i}$ and λ. In fact, $k_{i}=H_{1}($*ê*$\left(PK_{cc},sk_{i}r_{i}r_{cc}P_{0})\right)$ and $k_{i}^{\prime }=H_{1}($*ê*$\left(PK_{fd},sk_{i}r_{i}r_{fd}P_{0})\right)$ are computed by HID${}_{i}$. It is worth noting that the fog device only calculates $k_{i}^{\prime }=H_{1}\left(ê\left(PK_{i},sk_{fd}r_{fd}r_{i}P_{0}\right)\right)=H_{1}\left(ê\left(PK_{fd},sk_{i}r_{i}r_{fd}P_{0}\right)\right)$ at the privacy-preserving aggregated data generation phase and the control center only calculates $k_{i}=H_{1}\left(ê\left(PK_{i},sk_{cc}r_{cc}r_{i}P_{0}\right)\right)=H_{1}\left(ê\left(PK_{fd},sk_{i}r_{i}r_{cc}P_{0}\right)\right)$ at the privacy-preserving aggregated data decryption phase.

In the data aggregation stage, the aggregation operation by the fog device is performed in a ciphertext manner. For the control center, it only has the aggregated data $M=\frac{L(C^{\lambda }\;mod\;N^{2})}{L(g^{\lambda }\;mod\;N^{2})}\;mod\;N-\sum_{i=1}^{n}k_{i}$ and just gets the data sum $\sum_{i=1}^{n}m_{i}$. Even if an adversary has intruded into the control center database, privacy of a single device cannot be obtained. Like this, the individual sensing data privacy is still preserved.

### 4.2. Non-Repudiation and Unforgeability

In the proposed PDAF scheme, the private key is also used to sign the data packet to be sent by each entity before sending the message. Then, the data packet is verified based on the sender’s public key. Although the process can be realized by homomorphic signatures and the verification method used in database [50,51,52,53], the efficiency is very low. In PDAF, it is ensured that adversaries cannot forge a new signature by eavesdropping on signed messages and thus cannot implement forgery attacks. In other words, the entities’ private keys are properly kept by themselves, their messages sent has non-repudiation. Our program has the ability to discover the dishonest behavior of entities.

If the traditional one-to-one verification method is used, assuming that there are *k* signatures to be verified, a total of 2k bilinear pairing operations are required. To improve verification efficiency, we use a batch verification method. As described in step 1 of Section 3.2.4, *k* signatures are randomly assigned to equal-sized sets $S_{1}$ and $S_{2}$, where $|S_{1}|=\left\lfloor \frac{k}{2}\right\rfloor$, $|S_{2}|=\left\lceil \frac{k}{2}\right\rceil$. Then the signatures in $S_{1}$ and $S_{2}$ are respectively verified, that is

$$\begin{array}{c} ê\left(P_{0},\sum_{j\in S_{1}}\sigma_{j}\right)=\prod_{j\in S_{1}}ê(PK_{j},H_{2}(C_{j}∥ID_{INFORMATION}∥TS), \end{array}$$

$$\begin{array}{c} ê\left(P_{0},\sum_{j\in S_{2}}\sigma_{j}\right)=\prod_{j\in S_{2}}ê(PK_{j},H_{2}(C_{j}∥ID_{INFORMATION}∥TS). \end{array}$$

Based on the above batch verification method, the number of bilinear pairing operations is reduced from 2k to $2(\left\lfloor \frac{k}{2}\right\rfloor +1)$, and hence the efficiency of the algorithm is improved. Note that, the verification method can resist forgeries. For example, if the adversary aims to generate a forgery by computing

$$\sigma_{i}^{\prime }=\left\{\begin{array}{c} \sigma_{i}-a,\;i=1,2,\ldots ,\left\lfloor \frac{k}{2}\right\rfloor \\ \sigma_{i}+a,\;i=\left\lfloor \frac{k}{2}\right\rfloor +1,\left\lfloor \frac{k}{2}\right\rfloor +2,\ldots ,k. \end{array}\right.$$

In this case, the greatest probability that the adversary forges a valid signature is

$$C_{k/4}^{k/2}C_{k/4}^{k/2}=\frac{(k/2)!}{(k/4)!(k/2-k/4)!}\cdot \frac{(k/2)!}{(k/4)!(k/2-k/4)!}\cdot \frac{\left(k/2)!(k/2\right)}{k!}\;.$$

Obviously, when *k* is large enough, the above probability is negligible.

## 5. Performance Evaluation

In this section, the performance of the proposed PDAF scheme is evaluated in terms of the computation costs and communication overhead at the IoT devices, the fog device, and the control center.

### 5.1. Computation Cost

The proposed PDAF scheme achieves the privacy-preserving aggregation for hybrid IoT devices, in order to analyze this scheme more accurately, in terms of computation costs, we assume that there are *n* IoT devices associated with a fog device and will focus on measuring the time required for performing the cryptographic operations in the proposed scheme. where, we denote the computation costs of an exponentiation operation in $G_{1}$, an exponentiation operation in $G_{2}$, an exponentiation operation in $Z_{N^{2}}^{*}$, a multiplication operation in $Z_{N^{2}}^{*}$, a bilinear pairing operation and a Paillier decryption operation with $T_{e_{1}}$, $T_{e_{2}}$, $T_{e_{Z}}$, $T_{m_{Z}}$, $T_{p}$, $T_{pai}$, respectively.

For the control center, in order to generate a data collection request, CC needs to calculate $r_{cc}P_{0}$ and $\sigma_{cc}=sk_{cc}H_{2}(ID_{cc}∥ID_{fd}∥T_{s}∥r_{cc}P_{0}∥TS)$ which need $2T_{e_{1}}$ computation costs. In privacy-preserving aggregated data decryption phase, CC checks if $ê\left(P_{0},\sigma \right)=ê(PK_{cc},H_{2}(C∥ID_{fd}∥ID_{cc}∥T_{s}∥r_{fd}P_{0}∥TS)$, computers $k_{i}^{\prime }\;=\;H_{1}\left(ê\left(\;PK_{i},sk_{cc}r_{cc}r_{i}P_{0}\right)\right)$ and recovers the aggregated data *M* respectively involves $2T_{p}$, $T_{p}+T_{e_{2}}$ and $T_{pai}$ computation costs. Therefore, in time slot $T_{s}$, the computation cost for the control center is $3T_{p}+2T_{e_{1}}+T_{e_{2}}+T_{pai}$. For the fog device, it needs $\left(n+5\right)T_{p}+\left(n+1\right)T_{m_{Z}}+2T_{e_{1}}+2T_{e_{Z}}+T_{e_{2}}$ computation costs. Specifically, FD checks if *ê*$(\sigma_{cc},P_{0})=$*ê*$(H_{2}(ID_{cc}∥ID_{fd}∥T_{s}∥r_{cc}P_{0}∥TS)$ needs $2T_{p}+T_{e_{1}}$. After receiving all the Data_Rep of HID${}_{i},\left(1\leq i\leq n\right)$, the computation of the authenticity and integrity of nData_Rep based on batch verification involves $\left(n+2\right)T_{p}$. To compute $k_{i}^{\prime }=H_{1}\left(ê\left(PK_{i},sk_{fd}r_{fd}r_{i}P_{0}\right)\right)$, $C=\prod_{i=1}^{n}\left(C_{i}\cdot g^{-k_{i}^{\prime }}\right)\cdot H{\left(T_{s}\right)}^{\phi_{0}}\;mod\;N^{2}$ and $\sigma =sk_{fd}H_{2}(C∥ID_{fd}∥ID_{cc}∥T_{s}∥r_{fd}P_{0}∥TS)$, ($T_{p}+T_{e_{2}}$), ($\left(n+1\right)T_{m_{Z}}+2T_{e_{Z}}$) and $T_{e_{1}}$ are needed respectively. In PDAF, the computation costs for each hybrid IoT device is $2(T_{p}+T_{e_{2}}+T_{e_{Z}}+T_{e_{1}})$. In fact, the computation costs for the secret key $k_{i}=H_{1}($*ê*$\left(PK_{cc},sk_{i}r_{i}r_{cc}P_{0})\right)$, $k_{i}^{\prime }=H_{1}($*ê*$\left(PK_{fd},sk_{i}r_{i}r_{fd}P_{0})\right)$ involves $2(T_{p}+T_{e_{2}})$. To protect private information, HID${}_{i}$ needs $2T_{e_{Z}}$ computation costs for the ciphertext $C_{i}=g^{m_{i}+k_{i}+k_{i}^{\prime }}H{\left(T_{s}\right)}^{\phi_{i}}\;mod\;N^{2}$. To compute $\sigma_{i}=sk_{i}H_{2}(C_{i}∥ID_{i}∥ID_{fd}∥T_{s}∥r_{i}P_{0}∥TS)$, one $T_{e_{1}}$ is needed. We represent the computation costs in Table 1.

**Figure 3:**
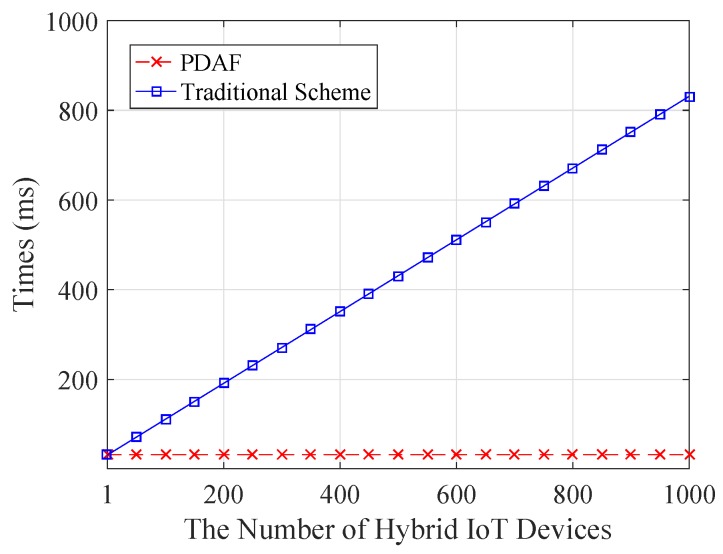
The computation cost comparison.

For the comparison with PDAF, in the following, we consider a traditional scheme, where all IoT devices blinded data $C_{i}$ are not aggregated into a ciphertext *C* by the fog device. Under this setting, for *n* IoT device data, the total computation cost of the control center will increase by $\left(n-1\right)T_{pai}$ over the PDAF. The computation comparison is shown in Figure 3. Obviously, our PDAF scheme largely reduces the computation cost for the control center.

In addition, in the security model of paper [10], a trusted third party is considered because the control center and fog devices are honest-but-curious which may be affected by malicious attacks. Based on the trusted third party, the security of the system is guaranteed, but the communication and computation overhead is high. In [30], although there is not a trusted third party, we find that the control center may be affected by undetected malwares and hence violates a single user’s data. It is possible to obtain sensitive information based on the private key λ, and the data aggregation scheme based on Paillier homomorphic encryption cannot completely protect sensitive information because the control center has the private key λ. In our proposed scheme, it is worth mentioning that the third-party trusted authority is not considered. In fact, the control center and fog device in PDAF are also honest-but-curious, but there is no risk of privacy leakage similar to [30].

### 5.2. Communication Overhead

**Figure 4:**
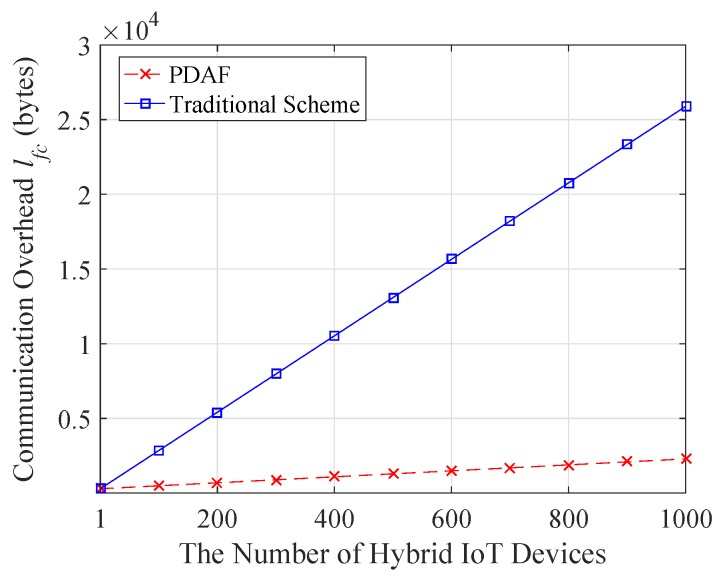
The communication overhead comparison.

In PDAF, we respectively denote the communication overhead of control center to fog devices (CC-to-FD), fog device to hybrid IoT devices (FD-to-HID), hybrid IoT devices to fog device (HID-to-FD) and fog device to control center (FD-to-CC) by $l_{cf},l_{fh},l_{hf}$, and $l_{fc}$. In addition, then, we define the size of each identity as 2 bytes, 4 bytes for $T_{s}$ or time stamp TS, the length of the Paillier ciphertext is 2048 bits. Let $G_{1}$ be a 160-bit elliptic curve and the length of the signature is 160 bits. Firstly, in the control center to fog device communication, the length of $Data\_Req=\{ID_{cc},ID_{fd},T_{s},r_{cc}P_{0},TS,\sigma_{cc}\}$ is 52 bytes, that is $l_{cf}=52$. In the fog device to hybrid IoT device communication, the Data_Req packet is of the form $\left\{ID_{fd},ID_{cc},T_{s},r_{fd}P_{0},r_{cc}P_{0},TS,\sigma_{cc}\right\}$ and $l_{fh}=72$. In the hybrid IoT device to fog device communication, the data collection request response Data_Rep of HID${}_{i}$(1≤i≤n) contains $C_{i},ID_{i},ID_{fd},T_{s},r_{i}P_{0},TS,\sigma_{i}$ and it length $l_{hf}=308$ bytes. To reduce the communication overhead, the aggregated signature and ciphertext are sent to the control center by the fog device, which only need 275 bytes. The response message is of the form $\left\{C,ID_{cc},ID_{fd},T_{s},{\left\{r_{i}P_{0}\right\}}_{1<i<n},TS,\sigma \right\}$ and the size is $l_{fc}=288+20n$ bytes where n is the number of hybrid IoT device. The communication overhead is listed in Table 2. Alternatively, if the traditional scheme is adopted, for *n* IoT device data, the length of $l_{fc}$ will increase to 288+256n bytes. As shown in Figure 4, we further show the change of the communication overhead with the hybrid IoT devices number *n*. It is shown that the PDAF scheme obviously reduces bandwidth usage and communication overhead for the FD-to-CC communication.

In summary, the proposed PDAF approach is privacy-preserving and efficient in terms of the computation cost and communication overhead.

## 6. Conclusions

In this paper, we have proposed a privacy-preserving data aggregation scheme based on the Paillier homomorphic encryption in fog computing and called PDAF. The idea realizes many security requirements such as privacy protection, non-repudiation, and unforgeability. The data aggregation technology based on homomorphic encryption not only can effectively protect the privacy of hybrid IoT devices but also can reduce the communication overhead of the system and improve the work efficiency of control centers and fog nodes. To improve the efficiency of data integrity checking, an efficient batch verification technology in use. In addition, blinding factor technology is also applied to our scheme, which makes the idea has better fault tolerance. Through analyzation of security and performance, the proposed scheme is reliable and efficient. 

## Figures and Tables

**Table 1:** The computation cost of PDAF.

	Computation Costs
CC	$3T_{p}+2T_{e_{1}}+T_{e_{2}}+T_{pai}$
FD	$\left(n+5\right)T_{p}+\left(n+1\right)T_{m_{Z}}+2T_{e_{1}}+2T_{e_{Z}}+T_{e_{2}}$
HID${}_{i}$	$2(T_{p}+T_{e_{2}}+T_{e_{Z}}+T_{e_{1}})$

**Table 2:** The communication overhead of PDAF.

	Communication Overhead (Bytes)
$l_{cf}$	52
$l_{fh}$	72
$l_{hf}$	308
$l_{fc}$	288+20n
